# Gamete donors' satisfaction; gender differences and similarities among oocyte and sperm donors in a national sample

**DOI:** 10.1111/aogs.12156

**Published:** 2013-06-24

**Authors:** Agneta Skoog Svanberg, Claudia Lampic, Ann-Louise Gejerwall, Johannes Gudmundsson, Per-olof Karlström, Nils-Gunnar Solensten, Gunilla Sydsjö

**Affiliations:** 1 Department of Women's and Children's Health, Uppsala UniversityUppsala, Sweden; 2 Department of Neurobiology, Care sciences and Society, Karolinska Institute StockholmStockholm, Sweden; 3 Institute of Clinical Sciences, Sahlgrenska AcademyGothenburg, Sweden; 4 Clintec, Department of Clinical Science, Intervention and Technology, Karolinska InstituteStockholm, Sweden; 5 Division of Obstetrics and Gynecology, Karolinska University HospitalStockholm, Sweden; 6 IVF Clinic, University Hospital of UmeåUmeå, Sweden; 7 Division of Obstetrics and Gynecology, Department of Molecular and Clinical Medicine Faculty of Health Sciences, Linköping UniversityLinköping, Sweden

**Keywords:** Ambivalence, mental health, oocyte donor, satisfaction, sperm donor

## Abstract

**Objective**To explore oocyte and sperm donors' emotional stress, experiences of care and satisfaction after donation.

**Design**Prospective multicenter study.

**Settings**All fertility clinics performing gamete donation in Sweden during the period 2005 to 2008.

**Population**Of 220 eligible oocyte donors who were approached, 181 agreed to complete the first questionnaire and 165 completed the second questionnaire 2 months after oocyte donation. Of 156 eligible sperm donors 119 accepted to complete the first questionnaire before donation. Eighty-nine participants completed the second questionnaire 2 months after sperm donation.

**Methods**Standardized and study-specific questionnaires.

**Main outcome measures**Satisfaction with the donation, respondents' mental health and overall care.

**Results**A larger percentage of sperm donors (97.8%) were satisfied with their overall experience of being a donor than oocyte donors (85.9%, *p *= 0.003). Some oocyte and sperm donors did not receive sufficient information about practical issues (9.1% and 13.5%, respectively) and future consequences (12.8% and 3.4%, respectively, *p* = 0.014). The donors' symptoms of anxiety and depression did not show any differences in relation to negative or positive perceptions of satisfaction. The donors who did not indicate ambivalence before treatment were on average almost five times more satisfied compared with those who did indicate ambivalence (odds ratio 4.71; 95% CI 1.34–16.51).

**Conclusions**Most donors were satisfied with their contribution after the donation. Oocyte and sperm donors who expressed ambivalence before donation were less satisfied after donation. In vitro fertilization staff fulfilled most of the donors' needs for information and care.

Please cite this article as: Skoog Svanberg A, Lampic C, Gejerwall A-L, Gudmundsson J, Karlström P-O, Solensten N-G, Sydsjö G. Gamete donors’ satisfaction; gender differences and similarities among oocyte and sperm donors in a national sample. Acta Obstet Gynecol Scand 2013; 92:1049–1056.

**Key Message**

The vast majority of Swedish gamete donors are satisfied with their contribution after donation. The oocyte and sperm donors who expressed ambivalence before the donation were less satisfied 2 months after donation.

## Introduction

Third-party conception would not be possible without a gamete supply from oocyte and semen donors. Previous studies have shown gender distinctions between oocyte and sperm donors 1–5. Women have appeared to be more involved in the process and outcome of the donation than their male counterparts 6. Oocyte donors seem more often found to be motivated by empathy towards the infertile couple, whereas sperm donors are more likely to be curious about their own fertility 4. In addition, results from earlier international studies on oocyte donors have shown that the higher the level of pre-donation altruistic motivation, the higher the post-donation satisfaction 7,8. In contrast, higher levels of pre-donation procedure-related ambivalence among oocyte donors have been associated with lower levels of post-donation satisfaction 8. In addition, women who believed that others would support them were more likely to report previous intention to donate than women who felt they would not be supported 9–13.

Presentation of information in terms of gains and losses can be powerful and could potentially influence an individual's preferences and decision-making process 14. Oocyte donation entails greater personal costs and medical risks than semen donation because the oocyte donors are exposed to possible physical risks as well as emotional and psychological burdens. In addition, the level of donor satisfaction is dependent on multiple factors, including time required, level of personal inconvenience, actual donation experience, and follow-up care 3,8,15,16. It has also been 17 reported that oocyte donors' attitudes towards various clinical scenarios change following donation, reflecting an overall expression of having greater reservations following the donation process. Other studies suggest that oocyte donors might not be aware of or consider in depth the broader implications of being a donor 2 and that counseling increases the donor's insight into the potential impact of gamete donation 10,15,18.

Anecdotal reports had suggested that those who choose to be donor candidates had experienced transient depression or anxiety to a greater degree than those who did not want to be donors 19 and had negative perceptions about the techniques involved 10,20. However, previous research has shown that oocyte donors tend to be positive about their donation experience, are psychologically well adjusted, that levels of satisfaction are high and that many would donate again 7,12,20–26.

There is clearly inadequate empirical information available to understand donors' emotional and psychological adjustment after donation. In a follow-up study, it has been reported that there was a lack of flexibility regarding anonymity and information about the outcome of donations 21. The author stated that improved donor satisfaction was likely to improve future donor recruitment and retention. To improve the readiness of the general population to act as gamete donors and given the relative importance and long-term impact of oocyte donation, more research on the psychosocial consequences for donors is needed.

Our aim for this study was twofold: first, to study differences in ambivalence before donation and symptoms of anxiety and depression in relation to satisfaction after donation between oocyte and sperm donors', and second, to study experiences of donor treatment and perception of donation.

## Material and methods

The Swedish multicenter study on gamete donation is a prospective longitudinal study of donors and recipients of donated gametes, including two comparison groups, carried out by collecting data from all fertility clinics performing gamete donation in Sweden, i.e. at the university hospitals in Stockholm, Gothenburg, Uppsala, Umeå, Linkoping, Örebro, and Malmö. During 2005–2008 gamete donors were approached regarding participation.

Donors were recruited through advertisements in local newspapers, blood donation programs, family/friends, and former oocyte donors. Potential donors spoke with the donor nurse coordinator on the telephone, who gave an overview of the principles of donation including the medical procedure and the social consequences. At the clinic visit the potential donors completed a medical questionnaire to provide information about surgical, medical, gynecologic/obstetric, and social history. The coordinator then conducted the assessment of the donor motivation and qualifications for admittance to the program. Potential donors received written information about the donation process and then had an interview with the medical doctor who reviewed medication, time commitment, legal and ethical issues. Donors were queried as to whether they would or would not participate in the donor program. Donor candidates were then assessed and evaluated by the clinic psychologists. If approved, potential donors were registered.

All women and men accepted as donors of oocytes/sperm were approached and asked if they would be willing to participate in the study. The only exclusion was persons who did not speak and/or read Swedish. Participants completed two questionnaires: on acceptance and 2 months after donation. Participation was rewarded with gift vouchers (worth approximately 12€).

**Figure 1:**
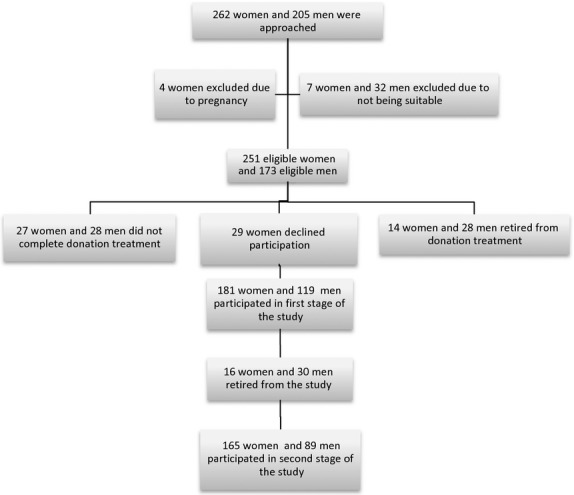
Flow diagram of participation of eligible oocyte donors.

Of 251 eligible oocyte donors, 41 withdrew from the treatment procedure and 29 declined participation, whereas 181 (86%) agreed and completed the first questionnaire. Of these, 165 (91%) also completed the second questionnaire (Figure 1). Of 173 eligible sperm donors, 54 withdrew from the medical procedure. Of 156 eligible sperm donors approached, 119 (76%) accepted and completed the first questionnaire (Figure 1). Eighty-nine (76%) completed the second questionnaire. Demographic data collected included age, highest stage of education, civil state, number of children, type of donation (anonymous/known to the recipient couples), and number of donations/treatments.

Ambivalence was measured with a modified Swedish version of an adapted Donor Ambivalence Scale for oocyte donors by Klock et al. 8. Permission was received from the author for using the scale in this study. The scale consists of seven multiple-choice items that measure mixed feelings about the donation. Responses are combined into a summary score between 0 and 7, with higher scores indicating greater ambivalence. In this study we considered a score ≥4 to indicate high ambivalence.

The Hospital Anxiety and Depression Scale (before and after donation), which measures anxiety and depressive symptoms, comprises 14 items (seven anxiety and seven depressive). Each scale was dichotomized into two levels, no depressive/anxiety symptoms (scores <8) and depressive/anxiety symptoms present (scores ≥8) 27.

Experience of donor treatment after donation was assessed using five study-specific questions about the care. The five response alternatives were collapsed into two categories “Not good at all/Not enough” to “Very good/Enough”.

Satisfaction with donation was measured after the donation with seven items developed on the basis of earlier research (3,8,20,25). In addition one single item assesses overall experience. The items were translated to Swedish, tested and found to be accurate in a pilot study. Each question was dichotomized into satisfied/not satisfied and a summary score computed. A score >6 was considered to mean individual satisfaction.

Perceptions of the donation were assessed using four study-specific questions. The response categories were formulated to indicate levels of agreement (“agree”, “neutral”, “disagree”, and “cannot form an opinion”).

The outcome of the donation, one single item was formulated on donation outcome: “Do you know the pregnancy result of the donation treatment?” The three response alternatives were “I do not know”, “the recipient was pregnant”, and “the recipient was not pregnant”.

### Statistical analysis

This was performed using IBM SPSS v.17 (Armink, NY, USA). Chi-squared testing was used to detect bivariate differences on the measurements of anxiety, depressive symptoms, experience, ambivalence, perception, and satisfaction in comparing oocyte and sperm donors. Anxiety and depressive symptoms were both dichotomized into whether anxiety/depressive symptoms were present or not, using the cut-off value of 8 (≥8 indicating anxiety/depressive symptoms).

Ambivalence was similarly defined into two levels indicating ambivalence or no ambivalence, as was perception and satisfaction. Binary logistic regression was used to evaluate the outcome satisfaction using ambivalence, anxiety, and depressive symptoms before and after treatment as predictors in three separate models. All models included background data such as age, education, and previous biological children. The study was approved by the Regional Ethics Review Board in Linköping (dnr M129-05-050223, T113-07 080122).

## Results

Sociodemographic data are presented in Table 1. The mean age was 30.4 years (standard deviation 4.5) for oocyte donors and 33.9 years (standard deviation 7.6) for sperm donors. Twenty-three (14%) oocyte donors and five (3%) sperm donors were so-called known donors, meaning that the recipient couple and the donors were known to each other. Of the donors, 156 (86%) knew persons that had infertility experience (data not shown).

**Table 1:** Sociodemographics of 165 oocyte donors and 89 sperm donors

	Oocyte donors		Sperm donors		
	*n*	%	*n*	%	*p*Value
Age					< 0.001
Mean, standard deviation	30.39	4.53	33.91	7.60	0.753
n≤30 years	69	41.8	35	39.8	
n>30 years	96	58.2	53	60.2	
Marital status					
Single	39	23.8	35	38.9	0.003
In a relationship	18	11.0	16	17.8	
Cohabitation/Married	107	65.2	38	42.2	
Education					
Elementary school	7	4.3	0	0.0	0.002
High school	81	49.4	29	32.2	
University	76	46.3	61	67.8	
Biological children					
No	110	67.5	31	35.2	< 0.001
Yes	53	32.5	57	64.8	
Donor known to the recipient couple					0.001
No	141	86.0	114	96.7	0.007
Yes	23	14.0	5	3.3	

The Hospital Anxiety and Depression Scale assessment of the oocyte donors before and 2 months after donation showed that 13/161 (8%) had anxiety before donation and 24/163 (15%) had anxiety after donation. For depressive symptoms there were 3/161 (2%) and 7/193 (4%) scoring above the cut-off level, respectively. Very few sperm donors showed depressive symptoms after the donation and no differences could be seen between oocyte and sperm donors (Table 2). The donors' anxiety and depressive levels did not show any differences in relation to negative or positive perceptions of satisfaction after the donation. The same results were found regarding satisfaction in relation to anxiety and depression levels after donation (data not shown).

**Table 2:** Oocyte donors' (*n* = 165) and sperm donors' (*n* = 89) symptoms of anxiety and depression before and after donation

	Oocyte donors		Sperm donors		
	*n*	%	*n*	%	*p*Value
Anxiety symptoms before donation					
No	148	91.9	83	93.3	0.703
Yes	13	8.1	6	6.7	
Anxiety symptoms after donation					
No	139	85.3	81	92.0	0.120
Yes	24	14.7	7	8.0	
Depression symptoms before donation					
No	158	98.1	88	98.9	0.655
Yes	3	1.9	1	1.1	
Depression symptoms after donation					
No	156	95.7	87	98.9	0.174
Yes	7	4.3	1	1.1	

Some donors thought that they did not receive sufficient information about practical issues and future consequences, but almost all (98%) were satisfied with their overall experience compared with oocyte donors (86%, *p *< 0.003) (Table 3).

**Table 3:** Oocyte donors' (*n*=165) and sperm donors'(*n*=89) experience of donor treatment

	Oocyte donors		Sperm donors		
	*n*	%	*n*	%	*p*Value
How did you experience during the first contact with the clinic at the time when you wanted to donate?					
Bad	14	8.5	7	7.9	0.853
Good	150	91.5	82	82.1	
What was your experience of meeting the staff at the clinic before the donation?					
Bad	5	3.0	8	9.0	0.041
Good	159	97.0	81	91.0	
Did you get enough information about practical issues regarding donation?					
Not enough	15	9.1	12	13.5	0.286
Enough	149	90.9	77	86.5	
Did you get enough information about future consequences regarding the donation?					
Not enough	21	12.8,	3	3.4	0.014
Enough	143	87.2	86	96.6	
How do you view your overall experience of the donation?					
Bad	23	14.1	2	2.2	0.003
Good	140	85.9	87	97.8	

Twenty-five (15%) oocyte donors reported a bad experience with being given hormones and 30 (18%) considered the oocyte retrieval painful. In general, most oocyte donors had a positive experience of donation (*n* = 130, 79%).

The donation had a positive impact on the lives of the donors (Table 4). There were no measured satisfaction differences between oocyte and sperm donors except that a higher percentage of oocyte donors regarded donation as a major event in their lives. A majority of donors (58%) did not know about the outcome of their donation; 93% of sperm donors vs. 44% of oocyte donors.

**Table 4:** Oocyte donors' (*n* = 165) and sperm donors' (*n* = 89) satisfaction with the donation

	Oocyte donors		Sperm donors		
	*n*	%	*n*	%	*p*Value
I am happy to help couples unable to have children by other means					
Agree	162	99.4	88	97.8	0.366
Neutral	0	0.0	1	1.1	
Disagree	1	0.6	1	1.1	
I feel as though I have made a contribution to my fellow human beings					
Agree	160	97.6	82	92.1	0.109
Neutral	3	1.8	4	4,5	
Disagree	1	0.6	3	3.4	
My life is more content					
Agree	79	48.2	49	55.7	0.473
Neutral	69	42.1	33	37.5	
Disagree	16	9.8	6	6.8	
I feel that I gave something away without receiving anything back					
Agree	12	7.5	6	6.7	0.105
Neutral	14	8.8	16	18.0	
Disagree	133	83.6	67	75.3	
This is the highlight (a major event) in my life					
Agree	60	37.0	25	27.8	0.021
Neutral	64	39.5	29	32.2	
Disagree	38	23.5	36	40.0	
I think I will brood about it for the rest of my life					
Agree	5	3.1	4	4.6	0.835
Neutral	13	8.1	7	8.0	
Disagree	143	88.8	76	87.4	

The perspectives of the oocyte donors as reported after donating differed from those of sperm donors (Table 5). Oocyte donors had a feeling of more support from family and friends compared with sperm donors. Despite this, a larger proportion of oocyte donors reported the donation to be completed.

**Table 5:** Oocyte donors' (*n* = 165) and sperm donors' (*n* = 89) perceptions post-donation

	Oocyte donors			Sperm donors					
	*n*	%	*n*	%	*p*Value
I am concerned over my future fertility					
Agree	13	7.9	5	5.6	0.796
Neutral	14	8.5	8	9.0	
Disagree	138	83.6	76	85.4	
I feel that my family and friends are proud of my donor contribution					
Agree	117	74.1	17	23.3	<0.001
Neutral	25	15.8	28	38.4	
Disagree	16	10.1	28	38.4	
It is hard for family and friends to understand all the aspects of my donation					
Agree	22	14.3	10	15.6	0.002
Neutral	25	16.2	24	37.5	
Disagree	107	69.5	30	46.9	
The donation is for me totally completed/finished after the donation procedure					
Agree	73	46.5	19	22.4	0.001
Neutral	23	14.6	21	24.7	
Disagree	61	38.9	45	52.9	

Responders aged 30 or more were approximately 60% less satisfied than those aged below 30 (Table 6). After adjustments of sociodemographic background variables, those who did not indicate ambivalence were almost five times more satisfied compared with those who did indicate ambivalence.

**Table 6:** Logistic regressions predicting satisfaction for 165 oocyte donors and 89 sperm donors

	Model 1 pre-anxiety/-depression		Model 2 post-anxiety/-depression		Model 3 ambivalence	
	OR	95% CI	OR	95% CI	OR	95% CI
Sperm donor	Reference level		Reference level		Reference level	
Oocyte donor	1.18	0.52–2.69	1.27	0.56–2.91	1.13	0.45–2.81
University	Reference level		Reference level		Reference level	
Elementary school	1.34	0.13–13.87	0.98	0.10–10.11	0.85	0.08–8.66
High school	1.36	0.65–2.85	1.34	0.64–2.78	1.17	0.54–2.55
Age, >30 years	Reference level		Reference level		Reference level	
Age, ≤ 30 years	0.43	0.18–1.01	0.41	0.18–0.97	0.46	0.19–1.12
Single	Reference level		Reference level		Reference level	
In a relationship	2.53	0.68–9.45	2.10	0.58–7.65	2.72	0.71–10.46
Cohabitation, married	2.74	0.97–7.72	2.36	0.87–6.37	2.43	0.85–6.98
Biological children, yes	Reference level		Reference level		Reference level	
Biological children, no	1.27	0.49–3.32	1.24	0.48–3.22	1.17	0.43–3.20
Pre-anxiety symptoms, yes	Reference level		–	–	–	–
Pre-anxiety symptoms, no	1.09	0.22–5.26	–	–	–	–
Pre-depression symptoms, yes	Reference level		–	–	–	–
Pre-depression symptoms, no	–	–	–	–	–	–
Post-anxiety symptoms, yes	–	–	Reference level		–	–
Post-anxiety symptoms, no	–	–	1.95	0.62–6.11	–	–
Post-depression symptoms, yes	–	–	Reference level		–	–
Post-depression symptoms, no	–	–	–	–	–	–
Ambivalence, yes	–	–	–	–	Reference level	
Ambivalence, no	–	–	–	–	4.71	1.34–16.51

## Discussion

In this national study of identifiable donors the vast majority were satisfied with their contribution after the donation. Those oocyte and sperm donors who expressed ambivalence before the donation reported less satisfaction after donation. This is in line with a previous study 8 showing a negative correlation between pre-donation ambivalence and post-donation satisfaction. In a recent Swedish study, sperm donors (39%) were found to be more ambivalent compared with oocyte donors (21%) before donation using the same ambivalence scale as the present study 4. In contrast, we found no gender differences in donor satisfaction post-donation in the present study.

Both oocyte and sperm donors were rather well educated. The sperm donors were more likely to have a university degree compared with oocyte donors. The demographic variables, emotional stress pre-donation and post-donation were not associated with the gamete donors' level of satisfaction with the donation. The level of anxiety and depressive symptoms of the oocyte and sperm donors were stable over time in the present study. This is in line with earlier studies of oocyte donors' characteristics 3,28.

There were positive attitudes to the overall experience of the donation. These results are in line with earlier studies reported in two reviews 12,26. Our previous studies on personality characteristics among both oocyte and sperm donors showed that they are autonomous, stable, and well adjusted 28,29. The results indicated that oocyte and sperm donors in general felt less worried, and suffered less from uncertainty and shyness. Purewal and van den Akker 30 have found that lower scores of perceived behavioral control in gamete donors predicted more willingness to donate. High levels of perceived behavior control predicted possible donation group and low levels of perceived behavior control predicted non-donation group in Purewal and van den Akker 30

The majority of the donors in the present study did not know the outcome of the donation; however, there were gender differences between the donor groups. Very few sperm donors knew the outcome of the donation. Kalfoglou and colleagues 18,21 found that 76% of anonymous oocyte donors were not told about the outcome but 75% of those donors wanted to know the outcome. Also, in two other studies 3,31 from the USA, the corresponding figures of the wish to know were the same. Our study of potential oocyte donors from the general population in Sweden 32 found that almost 50% said that they would not want information about the outcome of the donation. The level of interest of knowing the outcome of the donation could also reflect the motivation and satisfaction.

It may be hard for family and friends to fully understand and give support to donors. In our study the donation seems to be acknowledged more readily by the families and friends of oocyte donors than of semen donors, perhaps because for oocyte donors the “medical” intervention involved is less embarrassing to discuss with family and friends than the very much more private “masturbation” involved in sperm donation.

The seven clinics' practices concerning how the information is provided and choosing the potential donors are not identical but do follow the recommendations from the National Board of Health and Welfare. Policies concerning information on the results of the donation, i.e. pregnancies or number of children born, may differ in some respects between clinics.

When a program views a donor as a patient, rather than simply as a donor, the attitudes and experiences of each as a donor becomes a necessary component of care. This is probably more pronounced among oocyte donors because they go through medical interventions that place more demands on the oocyte donors than on sperm donors. It may seem simpler for sperm donors to make their donations, but even sperm donation is not a single event. The donors have to go through medical psychological screening, blood tests, and repeated semen donation. The time commitment and sometimes also the absence of financial incentives can make it very difficult to recruit gamete donors, although this is not always reported 1.

The main strength of the present study is the large sample size and that it is a prospective study including all fertility clinics performing gamete donation in Sweden. Distinct inclusion criteria and high response rates contribute to the external validity. However, no information is available about the donors who were not accepted or who chose not to participate in the present study, and it is possible that they have a different view of the questions asked.

In conclusion, the vast majority of donors expressed 2 months after donation that they were satisfied with their contribution. Oocyte and sperm donors who expressed ambivalence before the donation were less satisfied after the donation. There is a need for a longer follow-up to catch donor reflections about any offspring which might have future implications for them.
